# Application of the Stress and Anxiety to Viral Epidemics-6 (SAVE-6) and Coronavirus Anxiety Scale (CAS) to Measure Anxiety in Cancer Patient in Response to COVID-19

**DOI:** 10.3389/fpsyg.2020.604441

**Published:** 2020-11-23

**Authors:** Myung Hee Ahn, Jihoon Lee, Sooyeon Suh, Sangha Lee, Hwa Jung Kim, Yong-Wook Shin, Seockhoon Chung

**Affiliations:** ^1^Division of Psychiatry, Health Screening and Promotion Center, Asan Medical Center, Seoul, South Korea; ^2^Department of Psychiatry, Asan Medical Center, University of Ulsan College of Medicine, Seoul, South Korea; ^3^Department of Psychology, Sungshin Women’s University, Seoul, South Korea; ^4^Department of Clinical Epidemiology and Biostatistics, Asan Medical Center, University of Ulsan College of Medicine, Seoul, South Korea

**Keywords:** viral epidemics, COVID-19, cancer, mental health, anxiety

## Abstract

This study investigated the usefulness of the six-item Stress and Anxiety to Viral Epidemics (SAVE-6) scale and the Coronavirus Anxiety Scale (CAS) as tools to assess anxiety related to coronavirus disease (COVID-19) in cancer patients. A total of 221 patients with cancer responded to an anonymous online questionnaire between 15 July and 15 August 2020. The functional impairment of the patients was assessed using the Work and Social Adjustment Scale (WSAS), and the SAVE-6 and CAS were also applied. Among these 221 cancer patients, 110 (49.8%) had SAVE-6 scores ≥ 15 and 21 (9.5%) had CAS scores ≥ 5. Within the study population, 104 (47.1%) and 29 (13.1%) patients had WSAS scores ≥ 11 (moderate to severe functional impairment) and ≥ 21 (severe functional impairment), respectively. The correlations between the SAVE-6 and WSAS (*p* < 0.001) and CAS (*p* < 0.001) scores were statistically significant. The cut-off for the SAVE-6 was 15 points, while that for the WSAS was 11. Our results suggested that the SAVE-6 and CAS could be used to evaluate moderate and severe degrees of functional impairment related to mental health, respectively, in cancer patients during viral epidemics.

## Introduction

Coronavirus disease (COVID-19), which is caused by the novel severe acute respiratory syndrome coronavirus 2 (SARS-CoV-2) that originated in the Hubei province of China in December 2019, has spread rapidly worldwide (Chan et al., 2020) and been declared a global pandemic by the World Health Organization (World Health Organization [WHO], 2020). The widespread contagion and lockdown of the COVID-19 have caused considerable mental health problems such as anxiety, depression, and post-traumatic stress symptoms (Castelli et al., 2020; Talevi et al., 2020; Torales et al., 2020; Vindegaard and Benros, 2020). Different levels of psychological distress may appear depending on the healthcare workers (Di Tella et al., 2020; Lai et al., 2020; Li et al., 2020; Vindegaard and Benros, 2020), the elderly (Yang et al., 2020), and the presence of underlying disease (Wang et al., 2020).

Cancer diagnosis and treatment is a major stressor resulting in considerable mental health issues for patients, such as anxiety and depression (O’Hea et al., 2020). Compared with general populations, cancer patients are at a higher risk of mental health problems (Akechi et al., 1999; Massie, 2004; Ahn et al., 2015). Therefore, cancer patients may be more vulnerable to mental health problems.

In a recently published study of the cancer patients in China during the COVID-19 pandemic, patients reported adverse psychiatric symptoms with the highest percentages accounted for by depression, anxiety, and PTSD which were 23.4, 17.7, and 9.3%, respectively (Wang et al., 2020). Even in normal times, fear and anxiety affect the course of underlying diseases (Vanni et al., 2020). Patients with cancer undergoing active treatment or taking immunosuppressive medication are more vulnerable to viral infection (Kamboj and Sepkowitz, 2009; Gosain et al., 2020). Previous studies demonstrated the potential impacts of COVID-19-related anxiety on decision-making processes such as increased procedures and surgical refusal (Vanni et al., 2020), and chemotherapy postponement (Karacin et al., 2020). COVID-19 has caused oncology departments to act and adopt newly-devised guidelines for cancer care (Dinmohamed et al., 2020). Therefore, it is important to evaluate virus-related anxiety in cancer patients.

Despite the significance of viral epidemics, evaluating psychiatric symptoms without consideration of pandemic could limit its impact. Therefore, a specific instrument to measure mental health problems is needed in cancer patients under pandemic era. There are several scales that assess mental health problems related to viral epidemics. However, to our knowledge, there have been no studies validating these scales with the cancer patient sample.

The six-item Stress and Anxiety to Viral Epidemics (SAVE-6) (Chung et al., 2020), one of scales that measure the anxiety related to viral epidemics, is a self-report questionnaire. SAVE-6 was shown to sensitively detect people with non-pathological psychological problems and stresses (Ahn et al., 2020). It was validated among the healthcare workers sample, but not among the other samples. The Coronavirus Anxiety Scale (CAS) is a five-item self-report questionnaire that first published mental health screening tool to identify individuals with functional impairment due to COVID-19 (Lee, 2020a). Lee and researchers in other countries conducted a series of follow-up studies to validate the CAS and demonstrate its psychometric properties with solid reliability and validity (Evren et al., 2020; Lee, 2020b; Lee et al., 2020a,b). While developing and validating the SAVE-6 to examine the anxiety and worries regarding the coronavirus among cancer patients in South Korea, we also included the CAS to examine how these two measures can be applied to this population.

Considering the above, this study aimed to (1) screen for anxiety response to viral epidemics among cancer patients using the SAVE-6 and CAS, (2) exam the validity of the SAVE-6 among cancer patients, and (3) explore the appropriate cut-off score of the SAVE-6 as a useful tool for measuring the degree of functional impairment.

## Methods

### Participants

In the current pandemic era, this study administered a cross-sectional online survey to cancer patients who visited Asan Medical Center, Seoul, Korea, between 15 July and 15 August 2020. Asan Medical Center is one of the largest cancer centers in Korea. We advertised cancer patients who attended outpatient clinics of the Departments of Oncology, General Surgery, Radiation Oncology, and Asan Cancer Edu-Info Center regardless of their specific tumor were included consecutively. Inclusion criteria in addition to presence of a cancer disease were age 18 years and voluntary participation in the study. The subjects were able to see our advertisement in the hospital the study’s objectives, enrollment procedure, and the survey’s anonymous online design for measuring cancer patient’s anxiety in response to viral epidemics, and they were able to respond quickly by clicking the relevant link. The 221 responders completed this survey. The survey collected information on patient age, sex, and marital status and cancer type, illness duration, cancer stage, and current treatment modalities. The survey also included three additional questions: “Have you experienced or been treated for depression, anxiety, or insomnia?” (Currently under treatment, Have been treated in the past, Never been treat) to assess the past or current psychiatric history, “Do you feel that you are currently depressed or anxious, or do you need help for your mood state?” (Yes, No) to check patients’ current needs of being helped by a psychiatrist, and “Are you more afraid of coronavirus than cancer?” (No:0–Yes:10) to measure the severity of afraid of coronavirus than cancer in this pandemic era.

The subjects were provided an e-gift coupon valued at approximately 3 dollars for their participation. The study protocol was approved by the Institutional Review Board, which waived the requirement for written informed consent (No. 2020-1055).

### Rating Scales

#### SAVE-6 Scale

The SAVE-6, a self-administered, six-item questionnaire, assesses general anxiety response to viral epidemics. The SAVE-6 has been derived from the original SAVE-9 scale^1^ to assess work-related stress and anxiety responses of healthcare workers to the COVID-19 viral epidemic (Ahn et al., 2020). The SAVE-9 was clustered into two factors: (1) anxiety response to viral epidemics and (2) work-related stress. Factor 1 of the SAVE-9, termed SAVE-6, extracted for the general population. The scale includes six items: (1) Are you afraid the virus outbreak will continue indefinitely? (2) Are you afraid your health will worsen because of the virus? (3) Are you worried that you might get infected? (4) Are you more sensitive toward minor physical symptoms than usual? (5) Are you worried that others might avoid you even after the infection risk has been minimized? (6) Do you worry your family or friends may become infected because of you? Respondents rated their agreement with each item on five-point scales as follows: 0 (never), 1 (rarely), 2 (sometimes), 3 (often), and 4 (always). Previous study (Chung et al., 2020) used a cut-off score of the SAVE-6 was 15 based on the least mild degree (≥5 points) of the General Anxiety Scale-7 (GAD-7).

#### Coronavirus Anxiety Scale (CAS)

The CAS is a brief self-reported mental health screening tool for clinical anxiety and fear associated with the COVID 19 crisis or the so-called “coronaphobia” that was developed by Lee (2020a). This five-item scale measures dizziness, sleep disturbance, tonic immobility, appetite loss, and abdominal distress on a five-point scale from 0 (not at all) to 4 (nearly every day) based on symptoms over the past 2 weeks. The original CAS investigation showed solid reliability (α = 0.92) and validity in a sample of 775 adult residents in the United States. The optimized cut-off score of ≥ 9 (90% sensitivity and 85% specificity) discriminated between persons with and without dysfunctional anxiety. However, Lee’s replication study proposed to lower the CAS cut-off score to ≥5 for the general public as the sample in his original investigation comprised exclusively adults anxious about the coronavirus, whereas the sample in the replication study did not require experience related to COVID-19 (Lee, 2020b). The psychometric properties of the CAS have been verified in South Korea (Choi et al., 2020). Cronbach’s alpha for this study sample was 0.85.

#### Work and Social Adjustment Scale (WSAS)

The Work and Social Adjustment Scale (WSAS) is a self-reported scale of functional impairment due to an identified problem developed by Marks (Mundt et al., 2002) and widely used to quickly assess patients reporting functional impairment due to depression, anxiety, or alcohol misuse disorders. The current form of WSAS is a five-item scale that measures (1) the ability to work or study; (2) home management; (3) social leisure activities; (4) private leisure activities; and (5) the ability to maintain close relationships. Each item is rated on a nine-point Likert scale from 0 (not at all) to 8 (severely impaired). Total sum scores below 10 suggest a subclinical population, while scores ≥ 11 and ≥ 21 indicate significant but not severe and moderately severe to severe functional impairment, respectively (Mundt et al., 2002). For the purpose of our study, we used a Korean version of the WSAS that created and translated in previous studies (Lee, 2013; Choi et al., 2020), with the author’s permission.

### Statistical Analysis

All statistical analyses were performed using IBM SPSS Statistics for Windows, version 21.0 (IBM Corp., Armonk, NY, United States). The clinical characteristics were summarized as means ± standard deviation. For the corrections due to the occurrence of multiple comparisons among/between the SAVE-6, CAS, and WSAS, statistical significance was specified using a Bonferroni correction. Therefore, a two-tailed *p*-value of <0.0167 (which = 0.05/3 outcome variables) was defined as statistically significant. First, principal component analysis was done to explore the confirmatory factors of SAVE-6, and also performed. Second, Spearman’s correlation test was used to evaluate the correlation between SAVE-6 and other scales, since those scales scores were not normally distributed. And last, receiver operating characteristic (ROC) analysis was performed to determine the appropriate cut-off points of the SAVE-6 using the WSAS to explore the usefulness of the SAVE-6 as a functional impairment assessment tool.

## Results

The demographic characteristics of the study participants are summarized in Table 1. The mean age of the 221 patients who participated in this study was 50.1 ± 13.3 years. Of these, 168 (76%) patients were female and were younger than the male patients (48.7 ± 12.6 vs. 54.6 ± 14.9 years, *p* < 0.001). Breast (*n* = 89, 40.3%) was the most prevalent cancer followed by colorectal (*n* = 23, 10.4%), and gastro-esophageal (*n* = 20, 9.0%). Twenty (9.0%) patients had experienced or had been treated for depression, anxiety, or insomnia and 49 (22.2%) patients believed that they were depressed or anxious reported needing help for their mood state.

**TABLE 1:** Subjects’ demographic characteristics (*N* = 221).

**Variables**	
Sex (female)	168 (76.0%)
Age (years)	50.1 ± 13.3
Marital status (single/married)	39 (17.6%)/178 (80.5%)
Years from initial cancer diagnosis (years)	3.3 ± 3.5
**Cancer types**	
Breast	89 (40.3%)
Colorectal	23 (10.4%)
Gastro-esophageal	20 (9.0%)
Lung	15 (6.8%)
Pancreatic and biliary tract	11 (5.0%)
Head and neck	11 (5.0%)
Hematologic	9 (4.1%)
Liver	8 (3.6%)
Urinary tract	7 (3.2%)
Gynecologic	6 (2.7%)
Kidney	4 (1.8%)
Thyroid	4 (1.8%)
Others including dual diagnosis	14 (6.3%)
Cancer stages (0/I/II/III/IV/not confirmed)	7/40/53/40/37/44
**Current treatments**	
Chemotherapy	99 (44.8%)
Radiation therapy	69 (31.2%)
Hormone therapy	39 (17.6%)
No current treatment	48 (21.7%)
Surgical procedure within 3 months	47 (21.3%)
Have you experienced or been treated for depression, anxiety, or insomnia?	20 (9.0%)
Do you feel that you are currently depressed or anxious, or do you need help for your mood state?	49 (22.2%)
**Symptoms assessments**	
Stress and Anxiety to Viral Epidemic-6 (SAVE-6)	14.3 ± 4.4 (0–24)
Coronavirus Anxiety Scale (CAS)	1.2 ± 2.0 (0–11)
Work and Social Adjustment Scale (WSAS)	11.3 ± 8.9 (0–40)
Are you more afraid of coronavirus than cancer? (No:0–Yes:10)	3.4 ± 3.3 (0–10)

Among 221 cancer patients, 110 (49.8%) had SAVE-6 scores ≥ 15 (Chung et al., 2020), while only 21 (9.5%) had CAS scores ≥ 5 (Lee, 2020b). A total of 104 (47.1%) subjects had WSAS scores ≥ 11 (moderate to severe dysfunction) and 29 (13.1%) had WSAS ≥ 21 (severe dysfunction) (Mundt et al., 2002).

**FIGURE 1:**
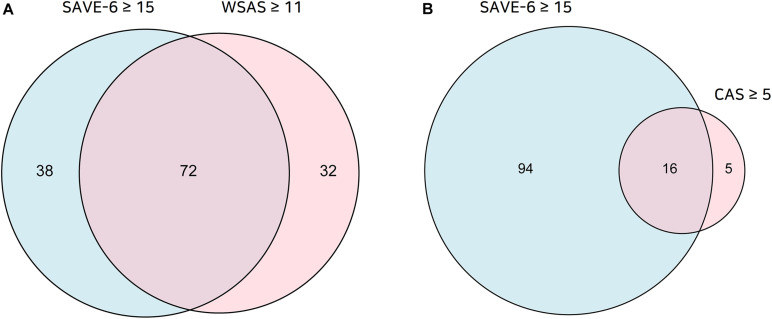
The proportion of participants with the SAVE-6 score ≥ 15 or WSAS score ≥ 11 **(A)** and SAVE-6 ≥ 15 or CAS score ≥ 5 **(B)**.

Principal component analysis revealed that all of 6 items in the SAVE-6 can be clustered into one factor (factor loading value 0.468–0.819). The Cronbach’s alpha of the six items was 0.804. We adopted one-factor structure based on the goodness-of-fit (Bartlett’s test of sphericity, *p* < 0.001; Kaiser–Meyer–Olkin = 0.795). The ROC analysis showed a SAVE-6 cut-off score of ≥15 for moderate to severe functional anxiety (≥11) based on the WSAS [area under the ROC curve (AUC) = 0.750, sensitivity = 0.69, specificity = 0.68]. The proportions of participants with various SAVE-6, WSAS, and CAS scores are shown in Figure 1.

The Spearman correlation test showed positively significant correlations between SAVE-6 scores and WSAS (rho = 0.48, *p* < 0.001), CAS (rho = 0.32, *p* < 0.001), and the visual analog score regarding feeling more afraid of coronavirus than cancer (rho = 0.17, *p* = 0.01).

## Discussion

In this study, we observed anxiety responses to the COVID-19 pandemic in half (49.8%) of cancer patients (SAVE-6 ≥ 15), while 9.5% showed ‘dysfunctional anxiety’ (Lee, 2020a) associated with the COVID-19 crisis (CAS ≥ 5). Furthermore, 47.1 and 13.1% of cancer patients had moderate to severe (WSAS ≥ 11) and severe (WSAS ≥ 21) “functional impairment associated with mental disorder” (Mundt et al., 2002), respectively. The appropriate cut-off score of SAVE-6 was 15 for moderate to severe degrees of dysfunction (≥11) in the WSAS.

Our previous study on the development of the SAVE-9 scale among healthcare workers (Chung et al., 2020) identified a SAVE-9 scale score cut-off for mild general anxiety as a Generalized Anxiety Disorder-7 items (GAD-7) scale score of ≥5. We observed that 21 points in the SAVE-9 scale and 15 points in the anxiety subcategory of the SAVE-9 (SAVE-6) were appropriate for mild anxiety symptoms among healthcare workers. This study explored the appropriate SAVE-6 cut-off score for moderate functional impairment related to mental health among cancer patients, for which a cut-off of 15 points was appropriate. A total of 110 (49.8%) patients had SAVE-6 scores ≥ 15, while 104 (47.1%) had WSAS scores ≥ 11. The degrees of functional impairment in work, social and private leisure activities, home work, and social relations can sensitively reflect the changes and severity of mental illness (Pedersen et al., 2017). We demonstrated that the SAVE-6 could be used to assess viral epidemic-specific psychological status in subjects not screened by the GAD-7 and WSAS. With more than 47% of cancer patients being affected, this should not be overlooked as the COVID-19 pandemic has been on-going for more than 6 months.

For a CAS cut-off score of ≥ 5 points, 9.5% of cancer patients showed dysfunctional anxiety regarding COVID 19, a finding well-supported by Lee’s replication study (Lee, 2020b). Although the original study describing the CAS proposed a dysfunctional anxiety cut-off score of ≥ 9 points, the subsequent study suggested lowering this cut-off to ≥ 5 points, corresponding to a sensitivity and specificity of 71 and 74% respectively, which showed acceptable diagnostic value. This change was proposed mainly because the participants of the original study were exposed not only to media coverage of coronavirus for at least 1 h during the previous 2 weeks but also reported significant anxiety and fear regarding the COVID 19 outbreak in the early stages. The cut-off score was suggested to be lowered to ≥ 5 for the general public in South Korea, where the coronavirus has been better controlled than in the United States. This was consistent with 13.1% of cancer patients meeting the WSAS cut-off score of ≥ 21.

The reduced CAS cut-off score may be associated with the purpose and characteristic of the measure. While the CAS was designed to screen people with functional impairment caused by the coronavirus pandemic mainly through their somatic symptoms, the SAVE-6 was developed to identify cancer patient anxiety regarding COVID-19 based on their emotional states. Thus, a scale that measures physical symptoms may better screen people with more severe anxiety than those with just emotional states. Thus, it is plausible that among 221 cancer patients, 110 (49.8%) had SAVE-6 scores ≥ 15 (Chung et al., 2020), while only 21 (9.5%) had CAS scores ≥ 5.

This study has the biases inherent to anonymous online questionnaires. Although face-to-face clinical interviews may have been helpful to evaluate psychiatric symptoms, it was not easy to interview cancer patients during the pandemic era with minimal contact. Additionally, sampling of our study was voluntary. The responses might also be biased, as this study utilized a self-reported survey. Older populations (greater than 65 years) were underrepresented in this online-based survey and most participants were women and breast cancer patients from one hospital in South Korea. Therefore, the generalizability of the results of this study to all patients with cancer may be limited.

## Conclusion

The SAVE-6 scale can be applied to cancer patients to measure moderate functional impairment related to mental health due to anxiety concerning the COVID-19 outbreak. The CAS scale, as suggested by the original developer (Lee, 2020a), can be applied to cancer patients to assess severe functional impairment related to mental health.

## Data Availability Statement

The raw data supporting the conclusions of this article will be provided, if requested.

## Ethics Statement

The studies involving human participants were reviewed and approved by the Asan Medical Center, Institutional Review Board, No. 2020-1055. The patients/participants provided their written informed consent to participate in this study.

## Author Contributions

MHA, JL, and SC conceived the study. SC obtained ethic approval. MHA and SC recruited participants and obtained data. YWS, SS, and SL organized the database. HJK performed statistical analyses.

## Conflict of Interest

The authors declare that the research was conducted in the absence of any commercial or financial relationships that could be construed as a potential conflict of interest.

## Footnotes

1 www.save-viralepidemic.net

## References

[B2] Ahn M. H. Shin Y. W. Suh S. Kim J. H. Kim H. J. Lee K. (2020). High work-related stress and anxiety response to COVID-19 among healthcare workers in South Korea: save study. *PsyArXiv* [Preprint] 10.31234/osf.io/9nxth PMC8544732 34478401

[B3] Ahn M. H. Park S. Lee H. B. Ramsey C. M. Na R. Kim S. O. (2015). Suicide in cancer patients within the first year of diagnosis. *Psychooncology* 24 601–607. 10.1002/pon.3705 25336020

[B4] Akechi T. Kugaya A. Okamura H. Nakano T. Okuyama T. Mikami I. (1999). Suicidal thoughts in cancer patients: clinical experience in psycho-oncology. *Psychiatry Clin. Neurosci.* 53 569–573. 10.1046/j.1440-1819.1999.00607.x 10595681

[B5] Castelli L. Di Tella M. Benfante A. Romeo A. (2020). The spread of COVID-19 in the Italian population: anxiety, depression, and post-traumatic stress symptoms. *Can. J. Psychiatry* 65 731–732. 10.1177/0706743720938598 32588644 PMC7502872

[B6] Chan J. F. Yuan S. Kok K. H. To K. K. Chu H. Yang J. (2020). A familial cluster of pneumonia associated with the 2019 novel coronavirus indicating person-to-person transmission: a study of a family cluster. *Lancet* 395 514–523. 10.1016/S0140-6736(20)30154-9 31986261 PMC7159286

[B7] Choi E. Lee J. Lee S. A. (2020). Validation of the Korean version of the obsession with COVID-19 scale and the Coronavirus anxiety scale. *Death Stud.* 10.1080/07481187.2020.1833383 [Epub ahead of print]. 34030606

[B8] Chung S. Kim H.J. Ahn M.H. Yeo S. Lee J. Kim K. (2020). Development of the stress and anxiety to viral epidemics-9 (SAVE-9) scale for assessing work-related stress and anxiety in healthcare workers in response to COVID-19. *PsyArXiv* [Preprint] 10.31234/osf.io/a52b4 PMC8648611 34873885

[B9] Di Tella M. Romeo A. Benfante A. Castelli L. (2020). Mental health of healthcare workers during the COVID-19 pandemic in Italy. *J. Eval. Clin. Pract.* 10.1111/jep.13444 [Epub ahead of print]. 32710481

[B10] Dinmohamed A. G. Visser O. Verhoeven R. H. A. Louwman M. W. J. van Nederveen F. H. Willems S. M. (2020). Fewer cancer diagnoses during the COVID-19 epidemic in the Netherlands. *Lancet Oncol.* 21 750–751. 10.1016/S1470-2045(20)30265-5 32359403 PMC7252180

[B11] Evren C. Evren B. Dalbudak E. Topcu M. Kutlu N. (2020). Measuring anxiety related to COVID-19: a turkish validation study of the coronavirus anxiety scale. *Death Stud.* [Epub ahead of print] 10.1080/07481187.2020.1774969 32490730

[B12] Gosain R. Abdou Y. Singh A. Rana N. Puzanov I. Ernstoff M. S. (2020). COVID-19 and cancer: a comprehensive review. *Curr. Oncol. Rep.* 22:53. 10.1007/s11912-020-00934-7 32385672 PMC7206576

[B13] Kamboj M. Sepkowitz K. A. (2009). Nosocomial infections in patients with cancer. *Lancet Oncol.* 10 589–597. 10.1016/S1470-2045(09)70069-5 19482247

[B14] Karacin C. Bilgetekin I. Basal F. B. Oksuzoglu O. B. (2020). How does COVID-19 fear and anxiety affect chemotherapy adherence in patients with cancer. *Future Oncol.* [Epub ahead of print]. 10.2217/fon-2020-0592 32677462 PMC7367513

[B15] Lai J. Ma S. Wang Y. Cai Z. Hu J. Wei N. (2020). Factors associated with mental health outcomes among health care workers exposed to coronavirus disease 2019. *JAMA Netw. Open* 3:e203976. 10.1001/jamanetworkopen.2020.3976 32202646 PMC7090843

[B16] Lee J. A. (2013). *The Effects of Cognitive Behavior Therapy for Nursing College Students with Irritable Bowel Syndrome.* Busan: Pusan National University.

[B17] Lee S. A. (2020a). Coronavirus anxiety scale: a brief mental health screener for COVID-19 related anxiety. *Death Stud.* 44 393–401. 10.1080/07481187.2020.1748481 32299304

[B18] Lee S. A. (2020b). Replication analysis of the Coronavirus anxiety scale. *Dusunen Adam J. Psychiatry Neurol. Sci.* 33 203–205. 10.14744/DAJPNS.2020.00079

[B19] Lee S. A. Jobe M. C. Mathis A. A. Gibbons J. A. (2020a). Incremental validity of coronaphobia: coronavirus anxiety explains depression, generalized anxiety, and death anxiety. *J. Anxiety Disord.* 74:102268 10.1016/j.janxdis.2020.102268 PMC7328548 32650221

[B20] Lee S. A. Mathis A. A. Jobe M. C. Pappalardo E. A. (2020b). Clinically significant fear and anxiety of COVID-19: a psychometric examination of the coronavirus anxiety scale. *Psychiatry Res.* 290:113112 10.1016/j.psychres.2020.113112 PMC7237368 32460185

[B21] Li Z. Ge J. Yang M. Feng J. Qiao M. Jiang R. (2020). Vicarious traumatization in the general public, members, and non-members of medical teams aiding in COVID-19 control. *Brain Behav. Immun.* 88 916–919. 10.1016/j.bbi.2020.03.007 32169498 PMC7102670

[B22] Massie M. J. (2004). Prevalence of depression in patients with cancer. *J. Natl. Cancer Inst. Monogr.* 32 57–71. 10.1093/jncimonographs/lgh014 15263042

[B23] Mundt J. C. Marks I. M. Shear M. K. Greist J. H. (2002). The work and social adjustment scale: a simple measure of impairment in functioning. *Br. J. Psychiatry* 180 461–464. 10.1192/bjp.180.5.461 11983645

[B24] O’Hea E. Kroll-Desrosiers A. Cutillo A. S. Michalak H. R. Barton B. A. Harralson T. (2020). Impact of the mental health and dynamic referral for oncology (MHADRO) program on oncology patient outcomes, health care utilization, and health provider behaviors: a multi-site randomized control trial. *Patient Educ. Couns.* 103 607–616. 10.1016/j.pec.2019.10.006 31753521 PMC7061075

[B25] Pedersen G. Kvarstein E. H. Wilberg T. (2017). The work and social adjustment scale: psychometric properties and validity among males and females, and outpatients with and without personality disorders. *Pers. Ment. Health* 11 215–228. 10.1002/pmh.1382 28681505

[B26] Talevi D. Socci V. Carai M. Carnaghi G. Faleri S. Trebbi E. (2020). Mental health outcomes of the CoViD-19 pandemic. *Riv. Psichiatr.* 55 137–144. 10.1708/3382.33569 32489190

[B27] Torales J. O’Higgins M. Castaldelli-Maia J. M. Ventriglio A. (2020). The outbreak of COVID-19 coronavirus and its impact on global mental health. *Int. J. Soc. Psychiatry* 66 317–320. 10.1177/0020764020915212 32233719

[B28] Vanni G. Materazzo M. Pellicciaro M. Ingallinella S. Rho M. Santori F. (2020). Breast cancer and COVID-19: the effect of fear on patients’ decision-making process. *In Vivo* 34 (3 Suppl.), 1651–1659. 10.21873/invivo.11957 32503825 PMC8378027

[B29] Vindegaard N. Benros M. E. (2020). COVID-19 pandemic and mental health consequences: systematic review of the current evidence. *Brain Behav. Immun.* 89 531–542. 10.1016/j.bbi.2020.05.048 32485289 PMC7260522

[B30] Wang Y. Duan Z. Ma Z. Mao Y. Li X. Wilson A. (2020). Epidemiology of mental health problems among patients with cancer during COVID-19 pandemic. *Transl. Psychiatry* 10:263. 10.1038/s41398-020-00950-y 32737292 PMC7393344

[B31] World Health Organization [WHO] (2020). *Coronavirus Disease (COVID-10) 2020.* Available online at: https://www.who.int/emergencies/diseases/novel-coronavirus-2019 (accessed August 15, 2020).

[B32] Yang Y. Li W. Zhang Q. Zhang L. Cheung T. Xiang Y. T. (2020). Mental health services for older adults in China during the COVID-19 outbreak. *Lancet Psychiatry* 7:e19 10.1016/S2215-0366(20)30079-1 PMC7128970 32085843

